# High preoperative CEA and systemic inflammation response index (C-SIRI) predict unfavorable survival of resectable colorectal cancer

**DOI:** 10.1186/s12957-023-03056-z

**Published:** 2023-06-09

**Authors:** Hao Cai, Yu Chen, Qiao Zhang, Yang Liu, HouJun Jia

**Affiliations:** Department of Gastrointestinal Surgery, The First Affiliated Hospital of Chongqing Medical University, No.1, Medical College Road, Yuzhong District, Chongqing, 400016 China

**Keywords:** Systemic inflammation response index, Colorectal cancer, Carcinoembryonic antigen, Prognosis

## Abstract

**Background:**

CEA and systemic inflammation were reported to correlate with proliferation, invasion, and metastasis of colorectal cancer. This study investigated the prognostic significance of the preoperative CEA and systemic inflammation response index (C-SIRI) in patients with resectable colorectal cancer.

**Methods:**

Two hundred seventeen CRC patients were recruited from Chongqing Medical University, the first affiliated hospital, between January 2015 and December 2017. Baseline characteristics, preoperative CEA level, and peripheral monocyte, neutrophil, and lymphocyte counts were retrospectively reviewed. The optimal cutoff value for SIRI was defined as 1.1, and for CEA, the best cutoff values were 4.1 ng/l and 13.0 ng/l. Patients with low levels of CEA (< 4.1 ng/l) and SIRI (< 1.1) were assigned a value of 0, those with high levels of CEA (≥ 13.0 ng/l) and SIRI (≥ 1.1) were assigned a value of 3, and those with CEA (4.1–13.0 ng/l) and SIRI (≥ 1.1), CEA (≥ 13.0 ng/l), and SIRI (< 1.1) were assigned a value of 2. Those with CEA (< 4.1 ng/l) and SIRI (≥ 1.1) and CEA (4.1–13.0 ng/l) and SIRI (< 1.1) were assigned a value of 1. The prognostic value was assessed based on univariate and multivariate survival analysis.

**Results:**

Preoperative C-SIRI was statistically correlated with gender, site, stage, CEA, OPNI, NLR, PLR, and MLR. However, no difference was observed between C-SIRI and age, BMI, family history of cancer, adjuvant therapy, and AGR groups. Among these indicators, the correlation between PLR and NLR is the strongest. In addition, high preoperative C-SIRI was significantly correlated with poorer overall survival (OS) (HR: 2.782, 95% CI: 1.630–4.746, *P* < 0.001) based on univariate survival analysis. Moreover, it remained an independent predictor for OS (HR: 2.563, 95% CI: 1.419–4.628, *p* = 0.002) in multivariate Cox regression analysis.

**Conclusion:**

Our study showed that preoperative C-SIRI could serve as a significant prognostic biomarker in patients with resectable colorectal cancer.

## Introduction

Globally, colorectal cancer (CRC) has become the third most common cancer and the second leading cause of tumor-related death. CRC accounts for approximately 10% of all new cancer diagnoses and cancer-related deaths each year [1, 2]. Although recent advances in pathophysiological research have provided more treatment options and personalized treatment regimens and significantly improved overall survival in patients with advanced disease, CRC is still responsible for nearly 900,000 deaths per year [2]. Therefore, it is necessary to develop comprehensive indicators to evaluate the prognosis of patients.

Malignancy is the product of multiple steps in which the acquisition of specific capabilities, such as evading growth suppressors and resisting cell death, ultimately combine to contribute to cancer’s growth, invasion, and metastasis. Among the contributing factors, tumor-associated inflammation is considered the seventh hallmark of cancer. Various factors, including smoking, chronic infections, environmental exposure such as silica and asbestos, and dietary habits, can lead to chronic inflammatory states in the host [3, 4]. Inflammatory cells can release some chemicals, especially reactive oxygen species, which prompt the genetic evolution of surrounding cancer cells in a highly malignant direction [5]. From an etiological standpoint, these chronic inflammatory states lead to host genetic and epigenetic alterations, resulting in 25% of malignancy cases [6]. Moreover, local and systemic inflammation leads to the release of biochemicals, including survival factors that limit cell death, certain enzymes that facilitate angiogenesis, and growth factors that sustain proliferative signaling, further promoting tumor progression [5, 7, 8].

Inflammation plays a critical role in all stages of colorectal cancer (CRC), with stages II, III, and IV exhibiting increased inflammation levels compared to stage I [9, 10]. Studies have found that acetyl heparinase levels in tumor tissue, particularly in stage II, are significantly elevated in CRC patients [10]. Heparanase, a protein involved in regulating the transcription of inflammation-related genes, participates in promoting the persistence of inflammation status, release of inflammatory cell extravasation, tumor-associated growth factors and cytokines, and acceleration of tumor growth [11, 12]. In addition, the inflammatory status is also present in non-tumor adjacent tissues of CRC [10]. The upregulation of COX2 levels regulated by NF-κB contributes to the persistence of inflammation, promoting angiogenesis, proliferation, and invasion of CRC tumor cells [13–15]. This suggests that non-tumor adjacent tissues may initiate a pro-tumor growth inflammatory status [10]. Therefore, tumor-associated inflammation significantly impacts CRC patients’ survival.

In summary, inflammation is a critical factor in the development and progression of CRC. The identification of specific biomarkers involved in tumor-associated inflammation could lead to the development of novel therapeutic strategies to improve the prognosis and treatment outcomes of CRC patients. Specific preoperative biomarkers can be utilized to evaluate systemic inflammation in CRC patients. Previous studies have explored the prognostic significance of various indicators, such as neutrophil-to-lymphocyte ratio (NLR) [16], lymphocyte-to-monocyte ratio (LMR) [17], platelet-to-lymphocyte ratio (PLR) [16], and C-reactive protein (CRP) [18]. In the preoperative phase, elevated levels of these biomarkers have been significantly associated with poorer survival. In recent years, a novel indicator has been proposed to assess systemic inflammation. The systemic inflammation response index (SIRI) was established based on neutrophils, monocyte, and lymphocyte. To date, the prognostic value of SIRI has been confirmed for various malignancies, such as pancreatic, cervical, and gastric cancers, with limited evidence indicating a significant association with survival outcomes in CRC patients [19–22]. In addition, carcinoembryonic antigen (CEA) is a common tool used to evaluate the prognosis of CRC, and high preoperative CEA levels predict poor disease-free survival (DFS), overall survival (OS), and increased risk of recurrence and metastasis, as shown in earlier studies [23, 24].

Therefore, we proposed a novel prognostic index based on CEA and SIRI (C-SIRI) to investigate whether it could accurately predict long-term survival in resectable CRC cases. Our aim was also to validate the prognostic value of SIRI in CRC patients and to provide research evidence for individual prediction and decision-making.

## Material and method

### Patients

From January 2015 to January 2017, a total of 300 CRC patients who underwent radical resection were consecutively enrolled at the First Affiliated Hospital of Chongqing Medical University (Chongqing, People’s Republic of China). The exclusion criteria were as follows: (1) people with a history of other primary malignant tumors or concurrent secondary malignancies; (2) neoadjuvant radiotherapy or radiochemotherapy ahead of surgery; (3) people with blood system diseases, infections, and treatments which influence the biomarkers; (4) people directly or indirectly die of diseases other than CRC; (5) people with emergent surgeries precede inclusion; (6) people with other serious diseases which have a great influence on life expectancy, such as intracerebral hemorrhage and myocardial infarction. Finally, 217 cases were included based on the criteria above. The study got approved by the independent ethics committee at The First Affiliated Hospital of Chongqing Medical University (K2023-104) and was conducted in line with the ethical standards of the World Medical Association Declaration of Helsinki.

### Data collection

The clinical information and blood indicators were obtained within 3 days prior to surgery, such as age, gender, past medical history, smoking and drinking history, family history, BMI, neutrophil, lymphocyte, monocyte, albumin, and carcinoembryonic antigen (CEA). Then, some composite metrics were calculated according to the following formulas: albumin-to-globulin ratio (AGR), neutrophil × monocyte-to-lymphocyte ratio (SIRI, systemic inflammation response index), serum albumin + 5 × lymphocyte (OPNI/PNI, Onodera’s Prognostic Nutritional Index), neutrophil-to-lymphocyte ratio (NLR), monocyte-to-lymphocyte ratio (MLR), platelet-to-lymphocyte ratio (PLR).

### Follow-up and treatment

For patients at high risk of recurrence and metastasis, adjuvant chemotherapy was offered based on the patient’s wishes. Trained subject members followed up all patients via telephone. Overall survival (OS) was defined as the period from pathological confirmation of cancer to the patient’s death or the most recent follow-up.

### Definition of CEA and SIRI

SIRI was calculated by the following equation: neutrophil × monocyte-to-lymphocyte ratio, and the optimal cutoff values for SIRI and CEA were obtained with x-tiles 3.6.1 software (Yale University, New Haven, CT, USA). Patients with low levels of CEA (< 4.1 ng/l) and SIRI (< 1.1) were assigned a value of 0, those with high levels of CEA (≥ 13.0 ng/l) and SIRI (≥ 1.1) were assigned a value of 3, and those with CEA (4.1–13.0 ng/l) and SIRI (≥ 1.1), or CEA (≥ 13.0 ng/l) and SIRI (< 1.1) were assigned a value of 2. Those with CEA (< 4.1 ng/l) and SIRI (≥ 1.1), or CEA (4.1–13.0 ng/l) and SIRI (< 1.1) were assigned a value of 1 (Table 1).

**Table 1:** CEA, SIRI, and C-SIRI scores

Scoring system	Value
CEA	
< 4.1 ng/l	0
4.1–13.0 ng/l	1
> 13.0 ng/l	2
SIRI	
< 1.1	0
≥ 1.1	1
Combination of CEA and SIRI	
CEA (< 4.1 ng/l) and SIRI (< 1.1)	0
CEA (< 4.1 ng/l) and SIRI (≥ 1.1)	1
CEA (4.1–13.0 ng/l) and SIRI (< 1.1)	1
CEA (4.1–13.0 ng/l) and SIRI (≥ 1.1)	2
CEA (≥ 13.0 ng/l) and SIRI (< 1.1)	2
CEA (≥ 13.0 ng/l) and SIRI (≥ 1.1)	3

*Abbreviations: SIRI* systemic inflammation response index, *CEA* carcinoembryonic antigen, *C-SIRI* CEA and systemic inflammation response index

### Statistical analysis

The optimal cutoff values of CEA and SIRI were obtained by x-tiles software (Yale University, Newhaven, Connecticut). Pearson’s *χ*^2^ test was utilized to reveal the correlation between variables. To find independent factors in the prognosis of colorectal cancer, hazard ratios (HRs) and 95% confidence intervals (CIs) were evaluated based on the univariate and multivariate Cox regression model. *P*-value less than 0.05 was statistical significance. All statistical analyses were carried out using SPSS 22.0 (SPSS IBM, Chicago, IL, USA).

## Results

### Patients’ characteristics

The study enrolled two hundred and seventeen patients (Table 2). The cohort included 124 (57.1%) male and 93 (42.9%) female patients. Based on the 8th edition of the AJCC staging criteria, most patients (76.5%) were diagnosed with stage II and III diseases, which accounted for an enormous proportion (90.7%) of the population with outcome events. During the follow-up period, a total of 54 patients had outcome events. In the patient cohort, 84 (38.7%) had a C-SIRI score of 0, 68 (31.3%) had a score of 1, 44 (20.3%) had a score of 2, and 21 (9.7%) had a score of 3. The proportion of patients with a preoperative C-SIRI score greater than or equal to 2 was 30%, and this group had half of the total outcome events.

**Table 2:** Baseline clinicopathological characteristics of patients with CRC

Variables	No. of patients (%)	No. of outcomes (%)
Gender		
Male	124 (57.1%)	36 (66.7%)
Female	93 (42.9%)	18 (33.3%)
Age (years)		
≥ 60	94 (43.3%)	43 (79.6%)
< 60	123 (56.7%)	11 (20.4%)
BMI		
≥ 24	58 (26.7%)	10 (18.5%)
< 18.5	20 (9.2%)	7 (13.0%)
18.5–23.9	139 (64.1%)	37 (68.5%)
Smoking		
No	127 (58.5%)	32 (59.3%)
Yes	90 (41.5%)	22 (40.7%)
Drinking		
No	142 (65.4%)	34 (63.0%)
Yes	75 (34.6%)	20 (37%)
Family history of cancer		
No	208 (95.9%)	51 (94.4%)
Yes	9 (4.1%)	3 (5.6%)
Tumor stage		
I	51 (23.5%)	5 (9.3%)
II	89 (41.0%)	22 (40.7%)
III	77 (35.5%)	27 (50.0%)
Tumor site		
Colon	90 (41.5%)	21 (38.9%)
Rectum	121 (55.8%)	29 (53.7%)
Others^a^	6 (2.8%)	4 (7.4%)
Adjuvant therapy		
No	73 (33.6%)	19 (35.2%)
Yes	144 (66.4)	35 (64.8%)
SIRI		
< 1.1	136 (62.7%)	25 (46.3%)
≥ 1.1	81 (37.3%)	29 (53.7%)
CEA (ng/l)		
< 4.1	114 (52.5%)	25 (46.3%)
4.1–13.0	68 (31.3%)	14 (25.9%)
≥ 13.0	35 (16.1%)	15 (27.8%)
C-SIRI		
0	84 (38.7%)	17 (31.5%)
1	68 (31.3%)	10 (18.5%)
2	44 (20.3%)	18 (33.3%)
3	21 (9.7%)	9 (16.7%)

*Abbreviations: BMI* body mass index, *SIRI* systemic inflammation response index, *CEA* carcinoembryonic antigen, *C-SIRI* CEA and systemic inflammation response index

^a^Included cecum and the junction of the rectum and sigmoid colon

### Correlation between preoperative SIRI and other variables

**Fig. 1:**
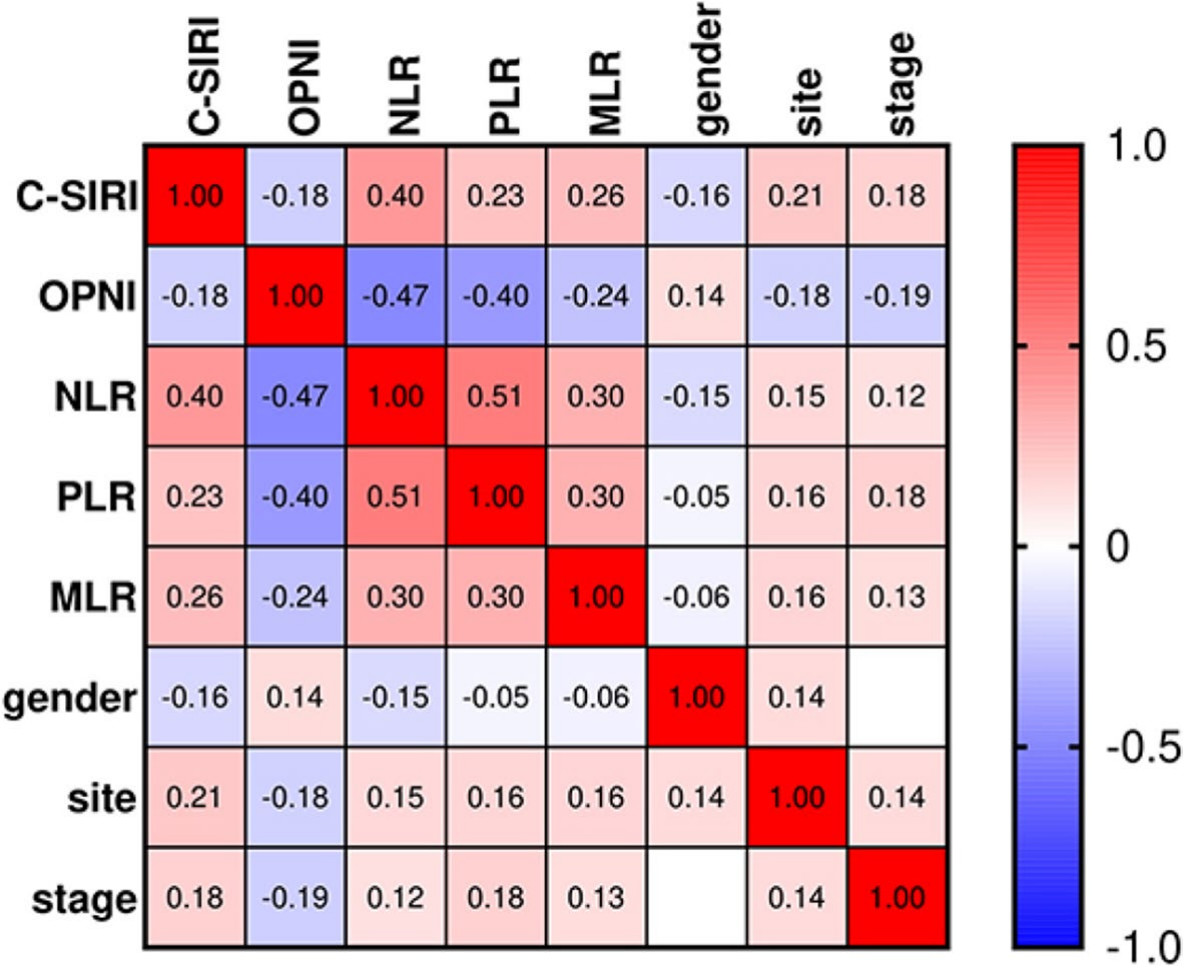
Correlation heat map of inflammation indicators. Abbreviations: *C-SIRI* CEA and systemic inflammation response index, *OPNI* Onodera’s prognostic nutritional Index, *NLR* neutrophil-to-lymphocyte ratio, *PLR* platelet-to-lymphocyte ratio, *MLR* monocyte-to-lymphocyte ratio

Based on Pearson’s *χ*^2^ test, preoperative C-SIRI is correlated with gender, site, stage, CEA, OPNI, NLR, PLR, and MLR (*p* = 0.019, *p* = 0.002, *p* = 0.009, *p* < 0.001, *p* = 0.06, *p* < 0.001,* p* = 0.001, and *p* < 0.001, respectively). However, there was no significant difference in age, BMI, family history of cancer, or adjuvant therapy groups. The heat map provided the correlation between eight indicators, reflecting the value and the shade of color (Fig. 1). Some strong correlations were observed. C-SIRI was negatively correlated with OPNI and gender and positively correlated with NLR, PLR, MLR, site, and stage, in which the relationship with NLR was relatively strong. Among eight indicators, the correlation between PLR and NLR is the strongest.

### Prognostic impact of preoperative CEA or SIRI

**Fig. 2:**
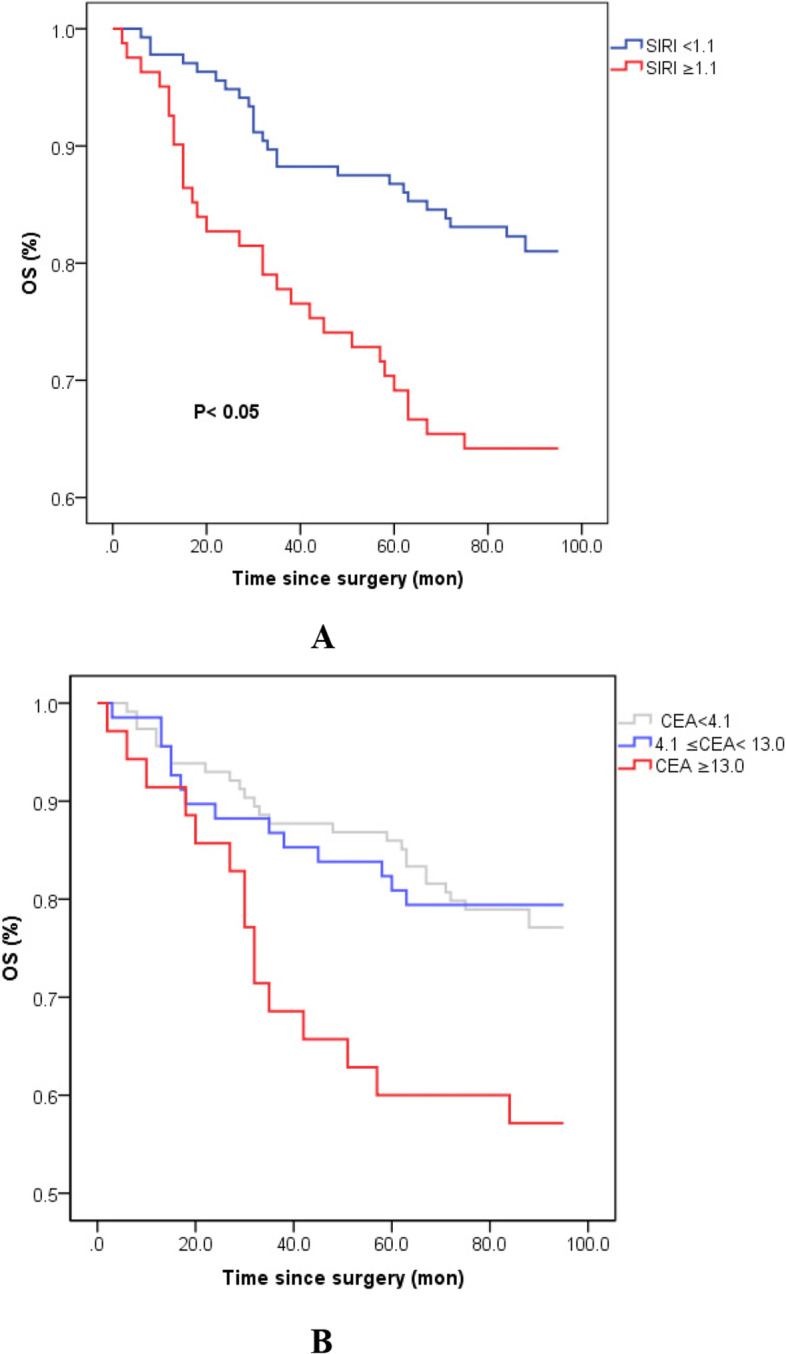
Kaplan–Meier survival curves of OS stratified by preoperative SIRI (**A**) and CEA levels (**B**) in 217 resectable colorectal cancer patients (with log-rank test). Abbreviations: *OS* overall survival, *CEA* carcinoembryonic antigen, *SIRI* systemic inflammation response index

The patients with relatively low CEA value (< 4.1 ng/l or 4.1–13.0 ng/l) had a significantly better OS than those with high CEA level (> 13.0 ng/l) (*p* = 0.009, 0.017, respectively, Fig. 2B). Although OS was not statistically significant between the low CEA (< 4.1 ng/l) and intermediate CEA groups (4.1–13.0 ng/l) (*p* = 0.548), there was still a trend towards better OS in the low CEA group than in the intermediate CEA group as shown in the Kaplan–Meier survival curves (Fig. 2B). Similarly, low SIRI level was associated with prolonged overall survival in resectable colorectal cancer patients (*p* = 0.02; Fig. 2A).

### Prognostic significance of preoperative C-SIRI in resectable colorectal cancer

**Fig. 3:**
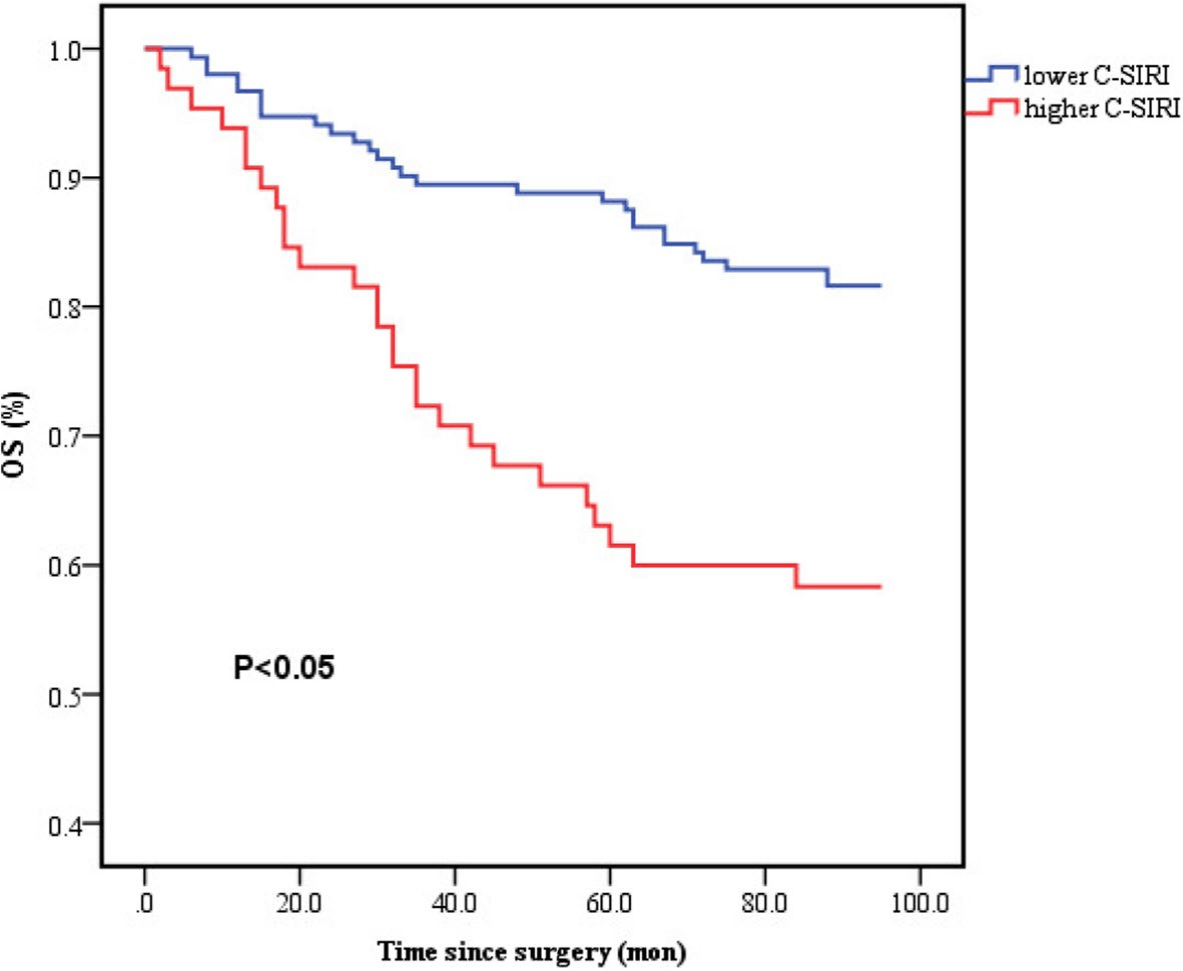
Kaplan–Meier survival curves of OS stratified by preoperative C-SIRI in 217 resectable colorectal cancer patients (with log-rank test). Abbreviations: *OS* overall survival, *C-SIRI* CEA and systemic inflammation response index. Notes: lower C-SIRI (score 0 or 1), higher C-SIRI (score 2 or 3)

Compared with higher C-SIRI levels, patients with lower C-SIRI tended to have a significantly better OS (*p* = 0.002, Fig. 3). The univariate and multivariate Cox models both showed that high C-SIRI was associated with an increased risk of OS. The HRs were 2.782 (95% CI: 1.630–4.746, *P* < 0.001) and 2.563 (95% CI: 1.419–4.628, *P* = 0.002), respectively. Additionally, preoperative C-SIRI (2/3 vs 0/1, HR: 2.563, 95% CI: 1.419–4.628,* P* = 0.002) remained an independent prognostic indicator for OS, based on multivariate analysis. At the same time, independent prognostic value for OS was found in age (< 60 vs ≥ 60 HR: 0.300, 95% CI: 0.150–0.600) and tumor stage (III vs I stage, HR: 5.392, 95% CI: 2.013–14.444). Furthermore, other parameters, including site, CEA, and SIRI, could also significantly predict OS (Table 3).

**Table 3:** Univariate and multivariate analysis for overall survival

Variables	Univariate analysis		Multivariate analysis	
	**HR (95%CI)**	***P***	**HR (95%CI)**	***P***
Gender (male vs female)	1.513 (0.859–2.663)	0.152	2.026 (0.945–4.344)	0.070
Age (years, < 60 vs ≥ 60)	0.285 (0.147–0.553)	< 0.01	0.300 (0.150–0.600)	0.001
BMI				
< 18.5	Ref	-	Ref	-
18.5–23.9	0.697 (0.311–1.563)	0.381	0.562 (0.240–0.319)	0.186
≥ 24	0.440 (0.167–1.156)	0.096	0.374 (0.126–0.958)	0.041
Smoking (yes vs no)	0.973 (0.565–1.674)	0.920	0.413 (0.179–0.952)	0.038
Drinking (yes vs no)	1.134 (0.653–1.971)	0.655	1.362 (0.609–3.045)	0.452
Family history of cancer (yes vs no)	1.371 (0.428–4.395)	0.595	1.523 (0.453–5.115)	0.496
Adjuvant therapy (yes vs no)	0.932 (0.533–1.628)	0.803	0.869 (0.476–1.588)	0.649
Tumor stage				
I	Ref	-	Ref	-
II	2.759 (1.045–7.286)	0.041	2.473 (0.908–6.738)	0.077
III	4.510 (1.736–11.718)	0.002	5.392 (2.013–14.444)	0.001
Tumor site				
Others^a^	Ref	-	Ref	-
Rectum	0.261 (0.091–0.747)	0.012	0.650 (0.195–2.164)	0.482
Colon	0.257 (0.088–0.753)	0.013	0.485 (0.150–1.570)	0.227
SIRI (< 1.1 vs ≥ 1.1)	0.446 (0.261–0.761)	0.003		NI
CEA				NI
< 4.1 ng/l	Ref	-		
4.1–13.0 ng/l	0.954 (0.496–1.836)	0.888		
≥ 13.0 ng/l	2.289 (1.206–4.345)	0.011		
C-SIRI score				
0/1	Ref	-	Ref	-
2/3	2.782 (1.630–4.746)	< 0.001	2.563 (1.419–4.628)	0.002

*Abbreviations: C-SIRI* CEA and systemic inflammation response index, *BMI* body mass index, *HR* hazard ratio, *CI* confidence interval, *SIRI* systemic inflammation response index, *CEA* carcinoembryonic antigen, *NI* not included, *Ref* reference

^a^Included the cecum and the junction of the rectum and sigmoid colon

## Discussion

To the best of our knowledge, the present study is one of few studies to establish a combined indicator that unites CEA and SIRI in resectable colorectal cancer. Our study showed that preoperative C-SIRI was significantly associated with clinical outcomes and possessed independent prognostic value. Patients with high preoperative C-SIRI had a poorer prognosis. Additionally, preoperative SIRI has predictive value for the survival of CRC patients.

Recent studies have highlighted the important role of systemic inflammation in tumor growth, proliferation, metastasis, and survival. It is a promising new direction for tumor treatment and monitoring. Several indicators have been reported to assess the level of systemic inflammation, such as NLR, PLR, MLR, LMR, and SIRI, and have been shown to have prognostic values in multiple tumors [25, 26]. Notably, SIRI was first proposed by Qi et al. as an independent prognostic marker in patients with pancreatic cancer [20]. Subsequent studies have affirmed the association of SIRI with survival in patients with liver, gastric, nasopharyngeal, and breast cancers [27–29]. Moreover, Cao et al. found that high preoperative SIRI was significantly associated with poorer OS and DFS of CRC patients, and predictive ability for the survival of CRC patients was superior to other inflammatory biomarkers, such as PLR, NLR, and systemic immune-inflammation index (SII) [22]. However, to date, multi-center data still need to confirm its relationship with CRC patients’ survival adequately.

In addition, CEA has first identified in fetal gut tissue and circulatory system of CRC patients over 50 years ago [30]. Subsequently, CEA was detected in the tumors from the gastrointestinal tract [31]. Despite the limited value of CEA for CRC screening, as elevated CEA levels may be due to some non-malignant diseases such as chronic inflammatory bowel disease, pancreatitis, and liver disease [32], substantial evidence has confirmed its predictive ability in the recurrence, metastasis, and survival of CRC patients. Becerra AZ and his colleagues found that elevated preoperative CEA levels were associated with a 62% increased risk of death compared to normal CEA levels [33], and the 5-year overall survival was 74.5% vs 63.4% [34]. Besides, Kim et al. suggested that elevated CEA level was expected to decrease exponentially after curative surgery, and survival was significantly better than that of patients with a sustained high level of CEA [35].

Therefore, we proposed that C-SIRI might predict accurately in resectable CRC patients. Our study results suggest that C-SIRI is statistically correlated with gender, site, stage, CEA., OPNI, NLR, PLR, and MLR. Among these factors, the strongest correlation was observed between PLR and NLR. However, no correlation was found between C-SIRI and BMI, adjuvant therapy, or AGR, indicating a need for further investigation. In addition, the patients with relatively low CEA value (< 4.1 ng/l or 4.1–13.0 ng/l) had a significantly better OS than those with high CEA level (> 13.0 ng/l) (*p* = 0.009, 0.017, respectively) based on log-rank test. However, no statistical difference in prognosis was found between the low CEA groups and the intermediate CEA groups (0.548), which may result from selection bias. Moreover, the study found that SIRI was a significant prognostic factor based on univariate analysis. Compared to the CRC patients with a low level of SIRI, those with SIRI ≥ 1.1 had a poorer prognostic outcome after curative resection, which is consistent with the prognostic value of SIRI in other malignancies. Multivariate analysis revealed that C-SIRI remained an independent prognostic predictor for OS (HR, 2.563, 95% CI, 1.419–4.628,* p* = 0.002). Thus, C-SIRI can serve as a reliable prognostic biomarker to support preoperative systemic inflammation response assessment and predict survival in CRC patients.

Although the present study has some limitations which should be considered, it provides valuable insights into the prognostic value of the novel indicator C-SIRI. Firstly, due to the limitation of specimen acquisition, the relationship between the local and systemic inflammation response, and its prognostic value, was not investigated. Secondly, as a single-center retrospective study, potential selection bias may exist. Thirdly, the small sample size calls for more research to support the findings. Therefore, multi-center studies with large samples and external validation are necessary for the future.

## Conclusion

In conclusion, the study showed that C-SIRI was significantly associated with OS in resectable CRC patients and confirmed its prognostic value based on univariate and multivariate analysis. This supports more accurate risk assessment and personalized treatment for resectable CRC patients.

## Data Availability

The datasets used in this study are available on request from the corresponding author.

## Abbreviations

CRC: Colorectal cancer
OS: Overall survival
DFS: Disease free survival
SIRI: Systemic inflammation response index
CEA: Carcinoembryonic antigen
C-SIRI: CEA and systemic inflammation response index
OPNI: Onodera’s prognostic nutritional Index
NLR: Neutrophil-to-lymphocyte ratio
PLR: Platelet-to-lymphocyte ratio
MLR: Monocyte-to-lymphocyte ratio
BMI: Body mass index
HR: Hazard ratio
CI: Confidence interval
NI: Not included
Ref: Reference
SII: Systemic immune-inflammation index

## References

[1] Sung H, Ferlay J, Siegel RL, Laversanne M, Soerjomataram I, Jemal A, Bray F (2021). Global cancer statistics 2020: GLOBOCAN estimates of incidence and mortality worldwide for 36 cancers in 185 countries. CA Cancer J Clin..

[2] Dekker E, Tanis PJ, Vleugels J, Kasi PM, Wallace MB (2019). Colorectal cancer. Lancet.

[3] Aggarwal BB, Shishodia S, Sandur SK, Pandey MK, Sethi G (2006). Inflammation and cancer: how hot is the link?. Biochem Pharmacol.

[4] Coussens LM, Werb Z (2002). Inflammation and cancer. Nature.

[5] Grivennikov SI, Greten FR, Karin M (2010). Immunity, inflammation, and cancer. Cell.

[6] Hussain P, Harris CC (2007). Inflammation and cancer: an ancient link with novel potentials. Int J Cancer.

[7] DeNardo DG, Andreu P, Coussens LM (2010). Interactions between lymphocytes and myeloid cells regulate pro- versus anti-tumor immunity. Cancer Metastasis Rev.

[8] Qian B-Z, Pollard JW (2010). Macrophage diversity enhances tumor progression and metastasis. Cell.

[9] Landskron G, De la Fuente M, Thuwajit P, Thuwajit C, Hermoso MA (2014). Chronic inflammation and cytokines in the tumor microenvironment. J Immunol Res.

[10] Alorda-Clara M, Torrens-Mas M, Hernández-López R, de la Ibarra Rosa JM, Falcó E, Fernández T (2022). Inflammation- and metastasis-related proteins expression changes in early stages in tumor and non-tumor adjacent tissues of colorectal cancer samples. Cancers.

[11] Vlodavsky I, Beckhove P, Lerner I, Pisano C, Meirovitz A, Ilan N (2011). Significance of heparanase in cancer and inflammation. Cancer Microenvironment.

[12] Masola V, Zaza G, Gambaro G, Franchi M, Onisto M (2020). Role of heparanase in tumor progression: molecular aspects and therapeutic options. Seminars in Cancer Biol-ogy.

[13] Wang S, Liu Z, Wang L, Zhang X (2009). NF-κB signaling pathway, inflammation and colorectal cancer. Cell Mol Immunol.

[14] Desai SJ, Prickril B, Rasooly A (2018). Mechanisms of phytonutrient modulation of Cy-clooxygenase-2 (COX-2) and inflammation related to cancer. Nutr Cancer.

[15] Sheng J, Sun H, Yu FB, Li B, Zhang Y, Zhu YT (2020). The role of cyclooxygenase-2 in colorectal cancer. Int J Med Sci.

[16] Zou ZY, Liu HL, Ning N, Li SY, Du XH, Li R (2016). Clinical significance of pre-operative neutrophil lymphocyte ratio and platelet lymphocyte ratio as prognostic factors for patients with colorectal cancer. Oncol Lett.

[17] Xiao W-W, Zhang L-N, You K-Y, Huang R, Yu X, Ding P-R (2015). A low lymphocyte-to-monocyte ratio predicts unfavorable prognosis in pathological T3N0 rectal cancer patients following total mesorectal excision. J Cancer.

[18] Zhou J, Wei W, Hou H, Ning S, Li J, Huang B (2021). Prognostic value of C-reactive protein, glasgow prognostic score, and C-reactive protein-to-albumin ratio in colorectal cancer. Front Cell Dev Biol..

[19] Huang H, Liu Q, Zhu L, Zhang Y, Lu X, Wu Y, Liu L (2019). Prognostic value of pre-operative systemic immune-inflammation index in patients with cervical cancer. Sci Rep.

[20] Qi Q, Zhuang L, Shen Y, Geng Y, Yu S, Chen H, Liu L, Meng Z, Wang P, Chen Z (2016). A novel systemic inflammation response index (SIRI) for predicting the survival of patients with pancreatic cancer after chemotherapy. Cancer.

[21] Li S, Lan X, Gao H, Li Z, Chen L, Wang W, Song S, Wang Y, Li C, Zhang H (2017). Systemic Inflammation Response Index (SIRI), cancer stem cells and survival of localised gastric adenocarcinoma after curative resection. J Cancer Res Clin Oncol.

[22] Cao Y, Zheng X, Hu Y, Li J, Huang B, Zhao N, Liu T, Cai K, Tian S. Levels of systemic inflammation response index are correlated with tumor-associated bacteria in colorectal cancer. Cell Death Dis. 2023;14: 10.1038/s41419-023-05602-9. 10.1038/s41419-023-05602-9 PMC9886998 36717544

[23] Tarantino I, Warschkow R, Worni M, Merati-Kashani K, Köberle D, M SB, A MS, Steffen T, Cerny T, Güller U. Elevated preoperative CEA is associated with worse survival in stage I–III rectal cancer patients. Br J Cancer. 2012;107:266–274. 10.1038/bjc.2012.267. 10.1038/bjc.2012.267 PMC3394990 22735902

[24] Forones NM, Tanaka M, Falcão JB (1997). CEA as a prognostic index in colorectal cancer. Sao Paulo Med J.

[25] Schiefer S, Wirsik, Naita Maren, Kalkum E, Seide SE, Nienhüser H, Müller B, Billeter A, Büchler MW, Schmidt T, Probst P. Systematic review of prognostic role of blood cell ratios in patients with gastric cancer undergoing surgery. Diagnostics. 2022;12:593. 10.3390/diagnostics12030593. 10.3390/diagnostics12030593 PMC8947199 35328146

[26] Zhu ZF, Zhuang LP, Zhang CY, Ning ZY, Wang D, Sheng J, Hua YQ, Xie J, Xu LT, Meng ZQ (2022). Predictive role of the monocyte-to-lymphocyte ratio in advanced hepatocellular carcinoma patients receiving anti-PD-1 therapy. Transl Cancer Res.

[27] Zhou Q, Su S, You W, Wang T, Ren T, Zhu L (2021). Systemic inflammation response index as a prognostic marker in cancer patients: a systematic review and meta-analysis of 38 cohorts. Dose-Response.

[28] Zhu M, Chen L, Kong X, Wang X, Fang Y, Li X, Wang J. The systemic inflammation response index as an independent predictor of survival in breast cancer patients: a retrospective study. Front Mol Biosci. 2022;9. 10.3389/fmolb.2022.856064. 10.3389/fmolb.2022.856064 PMC8918696 35295846

[29] Zeng X, Liu G, Pan Y, Li Y (2020). Development and validation of immune inflammation–based index for predicting the clinical outcome in patients with nasopharyngeal carcinoma. J Cell Mol Med.

[30] Gold P, Freedman SO (1965). Demonstration of tumor-specific antigens in human colonic carcinomata by immunological tolerance and absorption techniques. J Exp Med.

[31] Thomson DM, Krupey J, Freedman SO, Gold P (1969). The radioimmunoassay of circulating carcinoembryonic antigen of the human digestive system. Proc Natl Acad Sci.

[32] George PK, Loewenstein MS, O'Brien MJ, Bronstein B, Koff RS, Zamcheck N (1982). Circulating CEA levels in patients with fulminant hepatitis. Dig Dis Sci.

[33] Becerra AZ, Probst CP, Tejani MA, Aquina CT, González MG, Hensley BJ, Noyes K, Monson JR, Fleming FJ (2016). Evaluating the prognostic role of elevated preoperative carcinoembryonic antigen levels in colon cancer patients: results from the national cancer database. Ann Surg Oncol.

[34] Spindler BA, Bergquist JR, Thiels CA, Habermann EB, Kelley SR, Larson DW, Mathis KL (2017). Incorporation of CEA improves risk stratification in stage II colon cancer. J Gastrointest Surg.

[35] Kim JY, Kim NK, Sohn SK, Kim YW, Kim K, Hur H, Min BS, Cho CH (2009). Prognostic value of postoperative CEA clearance in rectal cancer patients with high preoperative CEA levels. Ann Surg Oncol.

